# Brightening of a dark monolayer semiconductor via strong light-matter coupling in a cavity

**DOI:** 10.1038/s41467-022-30645-5

**Published:** 2022-05-30

**Authors:** Hangyong Shan, Ivan Iorsh, Bo Han, Christoph Rupprecht, Heiko Knopf, Falk Eilenberger, Martin Esmann, Kentaro Yumigeta, Kenji Watanabe, Takashi Taniguchi, Sebastian Klembt, Sven Höfling, Sefaattin Tongay, Carlos Antón-Solanas, Ivan A. Shelykh, Christian Schneider

**Affiliations:** 1 Institute of Physics, Carl von Ossietzky University, Oldenburg, 26129 Germany; 2 Faculty of Physics, ITMO University, Saint-Petersburg, 197101 Russia; 3 Technische Physik, Universität Würzburg, Am Hubland, Würzburg, D-97074 Germany; 4 Institute of Applied Physics, Abbe Center of Photonics, Friedrich Schiller University, Jena, 07745 Germany; 5 Fraunhofer-Institute for Applied Optics and Precision Engineering IOF, Jena, 07745 Germany; 6 Max Planck School of Photonics, Jena, 07745 Germany; 7 Center for Nanoscale Dynamics (CeNaD), Carl von Ossietzky Universität Oldenburg, Carl-von-Ossietzky-Straße 9-11, 26129 Oldenburg, Germany; 8 School for Engineering of Matter, Transport, and Energy, Arizona State University, Tempe, 85287 AZ USA; 9 Research Center for Functional Materials, National Institute for Materials Science, 1-1 Namiki, Tsukuba, 305-0044 Japan; 10 International Center for Materials Nanoarchitectonics, National Institute for Materials Science, 1-1 Namiki, Tsukuba, 305-0044 Japan; 11 Science Institute, University of Iceland, Dunhagi 3, Reykjavik, IS-107 Iceland

**Keywords:** Two-dimensional materials, Photonic devices, Microresonators

## Abstract

Engineering the properties of quantum materials via strong light-matter coupling is a compelling research direction with a multiplicity of modern applications. Those range from modifying charge transport in organic molecules, steering particle correlation and interactions, and even controlling chemical reactions. Here, we study the modification of the material properties via strong coupling and demonstrate an effective inversion of the excitonic band-ordering in a monolayer of WSe_2_ with spin-forbidden, optically dark ground state. In our experiments, we harness the strong light-matter coupling between cavity photon and the high energy, spin-allowed bright exciton, and thus creating two bright polaritonic modes in the optical bandgap with the lower polariton mode pushed below the WSe_2_ dark state. We demonstrate that in this regime the commonly observed luminescence quenching stemming from the fast relaxation to the dark ground state is prevented, which results in the brightening of this intrinsically dark material. We probe this effective brightening by temperature-dependent photoluminescence, and we find an excellent agreement with a theoretical model accounting for the inversion of the band ordering and phonon-assisted polariton relaxation.

## Abstract (web summary)

Here, the authors show brightening of dark excitons by strong coupling between cavity photons and high energy, spin-allowed, bright excitons in monolayer WSe_2_. In this regime, the commonly observed photoluminescence quenching stemming from the fast relaxation to the dark ground state is prevented.

## Introduction

Atomically thin transition metal dichalcogenides (TMDCs) represent an emerging class of functional materials that are of particular interest in photonics due to their remarkable capability of efficient light emission and absorption^1–3^. In contrast to the vast majority of conventional semiconductors, their truly two dimensional (2D) nature results in the strong enhancement of the electron-hole Coulomb attraction, which makes their optical response to be dominated by exciton transitions even at room temperature^4–6^.

Since both the valence and the conduction bands emerge from the *p*-orbitals of the transition metals, the effects of spin-orbit coupling are of central importance in excitonic ordering. Specifically, the optical selection rules allow the excitation of the lowest energy exciton in monolayers of MoSe_2_ and MoTe_2_^7,8^, whereas in WSe_2_ and WS_2_ it is spin-forbidden, and thus the exciton transition remains optically dark^9^. As a consequence, at low temperatures, the luminescence of these dark materials is intrinsically quenched due to the fast relaxation of excitons into the dark ground state, and needs to be thermally ’activated’^10^. This creates a serious obstacle for optoelectronic applications which require maximized quantum efficiency, such as light-emitting diodes^2,11,12^, and conventional^13–15^—as well as polariton lasers^16,17^.

A number of methods to enhance the luminescence efficiency of optically dark 2D materials have already been investigated. These include the band-structure shaping by spin-orbit engineering^9^, the application of an in-plane magnetic field^7,18,19^, and the direct coupling of out-of-plane optical dipole moment of dark excitons with strongly localized electric fields in nanoantenna tips^20^, or a TM polarized optical mode at an oblique incidence^21^. However, the relevance of these approaches relying on high magnetic fields or nanoantenna tips is certainly limited for wide-spread use in applications.

In the present work, we follow an alternative approach to reach effective brightening, which is based on the idea that the nature of the exciton ground state of the system can be qualitatively changed in the strong light-matter coupling regime^22^, when optically active excitons are effectively hybridized with a spatially confined photonic mode of a microcavity, giving rise to composite elementary excitations known as exciton-polaritons^17,23,24^. Attempts to engineer the emission properties of organic molecules via the inversion of the singlet-triplet state configuration by strong light-matter coupling were reported recently^25,26^. However, for the cases studied thus far, the detailed analyses revealed that the reverse intersystem crossing rates mostly remain invariant even as the lower polariton energy is pushed below the triplet energy^26^, calling for further engineering efforts. We demonstrate the phenomena of the brightening by analyzing the temperature dependence of the photoluminescence (PL) from a WSe_2_ monolayer, showing that the trend of the rapid decrease of PL intensity with decreasing temperature is inverted if a WSe_2_ monolayer is placed inside a high finesse optical cavity. We support this remarkable experimental finding by theoretical modelling of the dynamics of the mode occupancies in both regimes.

## Results

### Experimental geometry and brightening mechanism

**Fig. 1:**
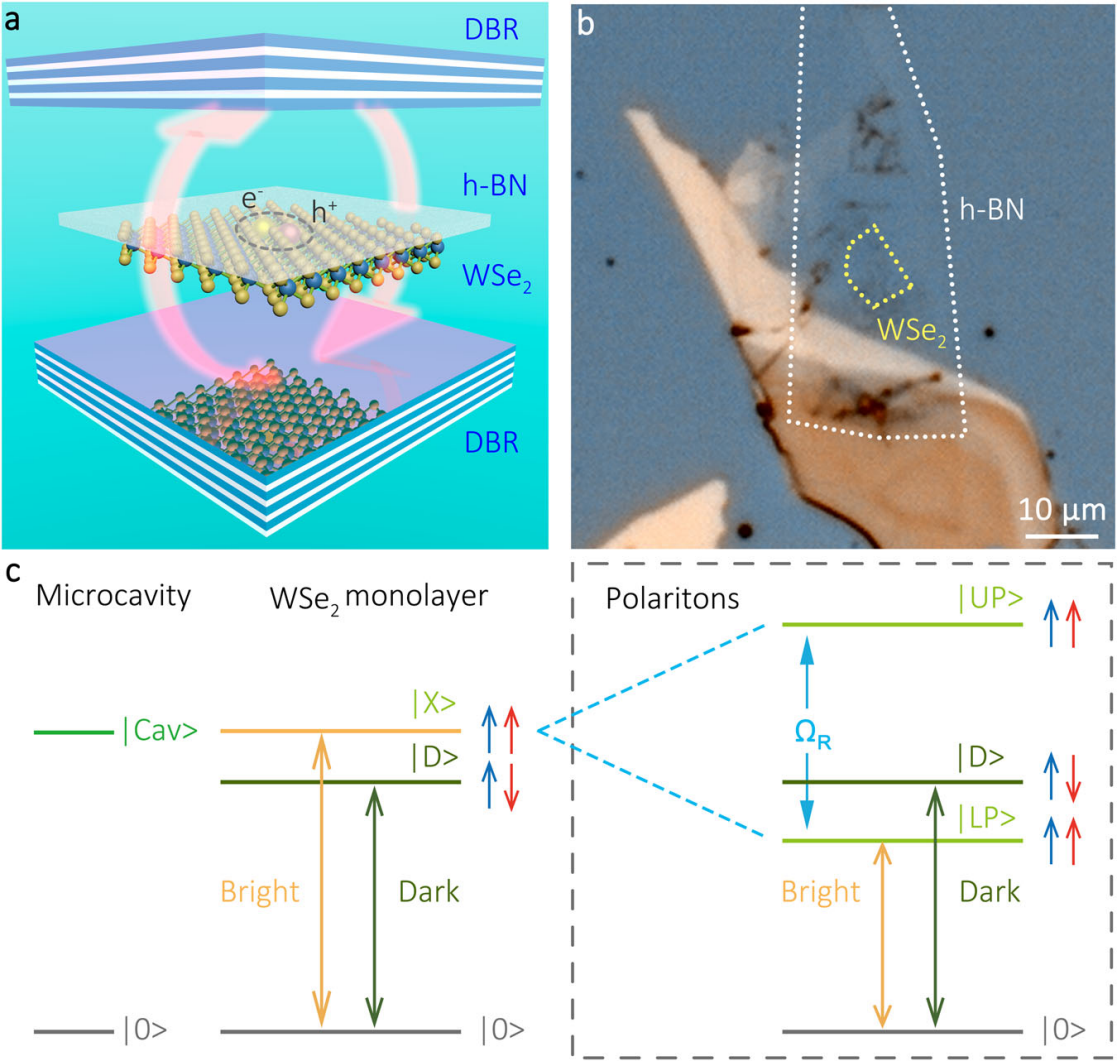
Sample structure and brightening mechanism. **a** Schematic illustration of sample structure with a WSe_2_ monolayer, capped with h-BN, and embedded between two dielectric DBRs. Two arrows represent the Rabi oscillation between excitons and microcavity photons. **b** Optical microscopic image of the sample. The WSe_2_ monolayer and h-BN boundaries are indicated with yellow and white dashed lines, respectively. **c** Scheme of WSe_2_ ground state brightening via strong coupling. In pristine WSe_2_ monolayers, the optically dark exciton $\left|D\right\rangle$ (brown line) is the lowest transition at the *K*-point, which lies ~40 meV below the bright exciton $\left|X\right\rangle$ (orange line). Exciton-polaritons are formed when optically bright excitons strongly couple to microcavity photons, the corresponding energy diagram is enclosed in a dashed box. The energy level of the resulting lower polaritons $\left|LP\right\rangle$ can be located below the dark exciton state $\left|D\right\rangle$, as long as the Rabi splitting Ω_*R*_ is sufficiently large. The lower polariton branch, which inherits the spin character from the bright exciton, becomes the ground state of the coherently dressed system. Thus, the band ordering is reversed, and the intrinsically dark 2D semiconductor is effectively brightened via the strong coupling with microcavity photons.

In Fig. 1a, we show the experimental system consisting of two distributed Bragg reflectors (DBRs) with a WSe_2_ monolayer flake situated in the antinode of the confined electromagnetic mode. The flake of WSe_2_ is mechanically exfoliated from a bulk crystal and it is capped with hexagonal boron nitrite (h-BN) via a deterministic dry-transfer method. Both DBRs are composed of SiO_2_/TiO_2_, the corresponding Bragg wavelength is 750 nm. An optical microscope image of the sample is displayed in Fig. 1b. The WSe_2_ monolayer flake, indicated with a dashed yellow line, forms a finite-size trap for exciton-polaritons, with a size of ~10 × 7  μm^2^.

The brightening mechanism in WSe_2_ monolayers in the strong light-matter coupling regime is illustrated in Fig. 1c. The sequence of the two lowest dark $\left|D\right\rangle$ and bright $\left|X\right\rangle$ exciton states at the *K*-point is given in the left panel. The spins of an electron and a hole in the ground state (brown line) are antiparallel to each other, and direct optical excitation of this singlet configuration is forbidden in the electric dipole approximation, making the ground state optically dark. The energy of the optically active triplet state (orange line) is ~40 meV higher^21,27^.

If a WSe_2_ monolayer is placed inside an optical microcavity, the strong light-matter coupling can dramatically reshape the energy spectrum of the system, as it is illustrated in the right panel of Fig. 1c. Indeed, bright excitons interact with cavity photons, giving rise to upper and lower polariton (UP and LP) modes. The Rabi splitting between these two hybrid modes Ω_*R*_, depends on the excitonic oscillator strength and cavity quality factor. If Ω_*R*_ is sufficiently large, one can push the LP energy below the dark exciton energy, reversing the energy level ordering: From one characteristic for an optically dark material, to a corresponding bright one. Note, that the LP energy can be tuned not only by changing the Rabi splitting but also by changing the relative detuning between the bare photon and exciton modes.

**Fig. 2:**
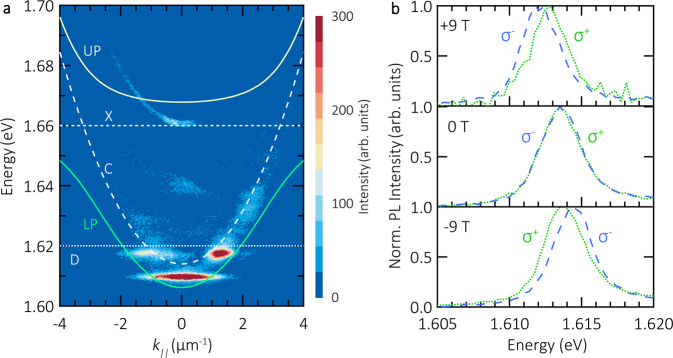
Polariton dispersion relation and valley-Zeeman effect. **a** Dispersion relation of exciton-polaritons at ambient conditions. The exciton (X) and microcavity photon (C) are represented by dotted and dashed lines, respectively. The dark exciton is marked as D. The upper and lower polariton (UP and LP) branches are plotted as solid lines. The discrete energy modes of LP are a typical dispersion relation of polaritons confined at a finite-size trap. **b** Normalized circularly polarized PL intensity spectra of exciton-polaritons under a magnetic field at room temperature. These spectra are extracted from the dispersion relations at zero in-plane momentum *k*_∥_ = 0. From top to bottom panels, the applied magnetic field is +9, 0 and −9 T, respectively. *σ*^+^ (*σ*^−^) denotes the emission of light with right (left)-hand circular polarization. An energy splitting is observed under the application of magnetic fields.

We use angle-resolved photoluminescence spectroscopy to study the optical properties of the coupled monolayer-microcavity system. The distribution of the PL intensity in energy and momentum is shown in Fig. 2a. The finite size of the flake results in the discretization of the energy levels of lower polaritons^28^, which yields a dispersion-less ground state at 1.610 eV. It furthermore yields a first excited state at 1.618 eV, with emission maxima at finite k-values, and finally a continuous emission band at ∣*k*_∥_∣ > 2 μm^−1^. The full quantitative description of the modes in connection with the precise shape of the monolayer is derived in ref. ^29^ by accounting the energy spectrum of polaritons in the presence of an external potential V(r) via numerically solving a Schrödinger equation for polaritons. The slightly asymmetric distribution of PL intensity at high energy results from the irregular geometry of WSe_2_ flake, as discussed in detail in ref. ^29^.

We notice, that the model which we apply in Supplementary section 2 is a simplified and less technical, yet analogous approach based on the discretization of the photon field as prior to the coupling to the excitons. In the model, the discretization is considered first in the photon field: due to the lateral confinement of the cavity and the flake, the photonic modes are essentially discretized. This discretization of photon modes then translates into the discretized polariton spectrum. Hence, this model is a reduced version of the full solution to the Schrödinger equation. It is important to point out, that these two formulations are equivalent and yield the same polaritonic ground and first excited states.

To fit experimental data, we use the two coupled oscillator model, with parameters extracted from the experimental data. The energies of the bright exciton (X) and cavity photon (C) are 1.660 eV and 1.614 eV, respectively, the Rabi splitting is 41  meV.

Due to the red-detuned conditions of our microcavity, and the fact that polaritons efficiently populate the ground-state of the polariton trap, a direct verification of strong coupling conditions via mapping of the Rabi-splitting is difficult. However, it is still possible to directly verify the strong coupling conditions of our TMDC-cavity system unambiguously by resorting to magnetic field measurements: in atomically thin TMDCs, the valley pseudospin is associated with magnetic moments^30,31^. Since exciton-polaritons inherit the spin from excitons, they also experience the valley-Zeeman effect, similar to bare excitons. In contrast, the effect is obsolete for cavity photons^32^, rendering magnetic measurement a powerful and elegant tool to distinguish hybrid exciton-polaritons from pure photonic modes.

In Fig. 2b, we plot circularly polarized components of the PL recorded in the external magnetic field. For the sake of clarity, both components have been normalized in their intensity. The slight energy offset between the two panels is induced by the reduction of air pressure in our experimental apparatus which renormalizes the cavity energy (see Supplementary Fig. S2 for more details). One clearly sees the energy splitting of ~0.5 meV between *σ*^+^ and *σ*^−^ polarized components in the magnetic field of +9 T (top panel), which unambiguously indicates the presence of the excitonic component. As expected, the sign of the Zeeman-splitting is changed if the direction of magnetic field is inverted (−9 T, bottom panel). It is worth noting, that the effect persists for weak pump powers, as shown in Supplementary Fig. S3. We have demonstrated that our sample exhibits macroscopic phase coherence^29^, which rules out the purely excitonic behaviour. Considering the sample simultaneously presents the Zeeman splitting and macroscopic phase coherence, its polaritonic origin is unambiguously proved.

### Manifestation of the ground state brightening

To demonstrate the brightening effect in the regime of the strong light-matter coupling, we compare the temperature-dependent PL for the isolated WSe_2_ flake and the flake placed inside the microcavity. The characteristic temperature-dependent exciton response has been employed previously to verify the conduction band inversion in MoWSe_2_ alloy monolayers^9^.

We first turn our attention to the investigation of the bare exciton response of a pristine WSe_2_. To warrant a valid comparison, the WSe_2_ monolayer is exfoliated from the same crystal and transferred on an identical DBR as that used in our polariton sample. Again, the monolayer is capped by a thin h-BN layer. Throughout our experiments, we used a non-resonant continuous-wave laser at 532 nm focused on a spot of ~3 μm diameter to excite the sample (see more details of the setup in the “Methods” section).

**Fig. 3:**
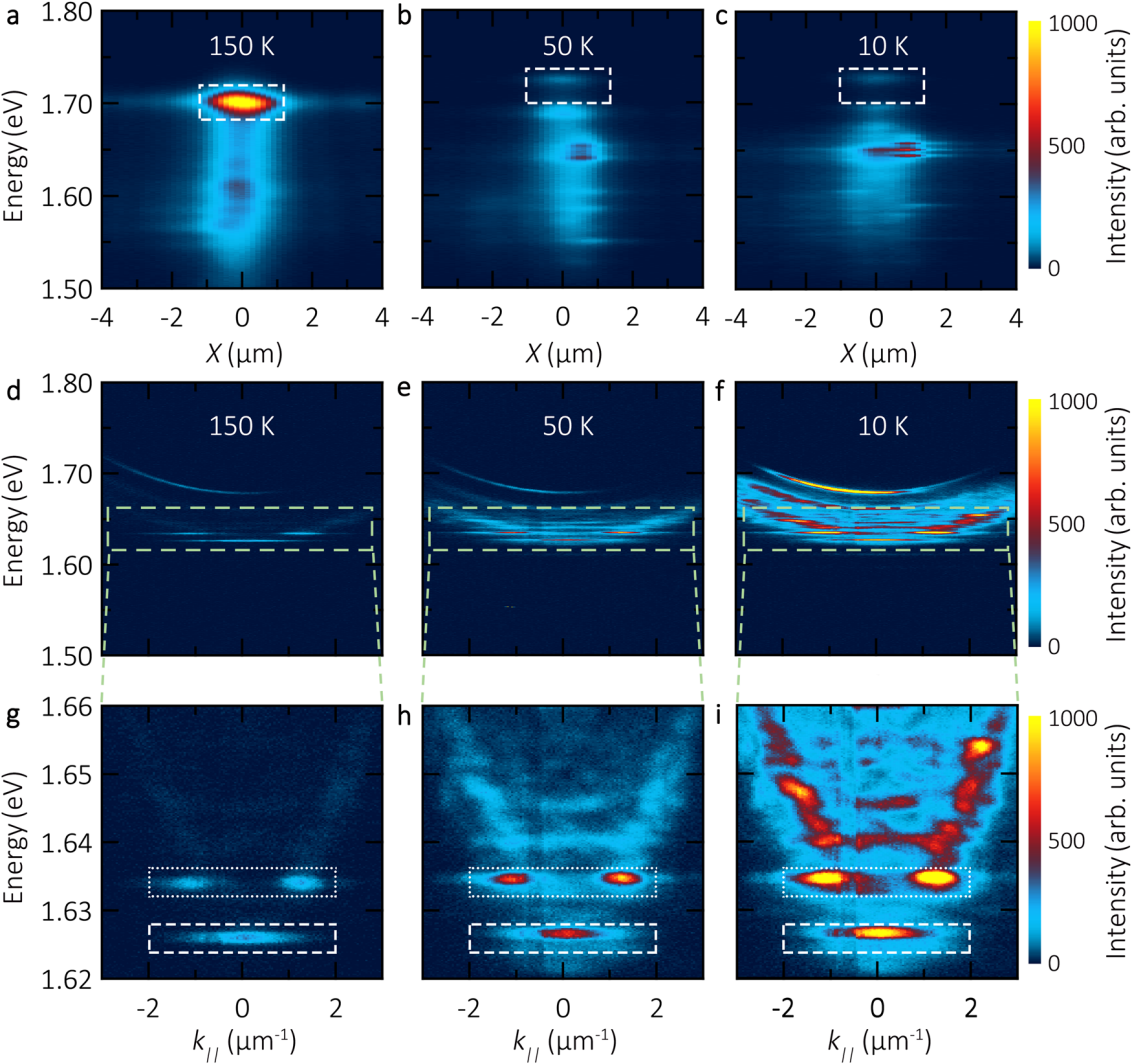
Temperature-dependent PL of bare excitons and exciton-polaritons. **a**–**c** Real-space resolved PL intensity distribution of a pristine WSe_2_ monolayer flake as a function of energy at temperatures 150, 50 and 10 K, respectively. The emission of bare excitons becomes dimmer as temperature decreases: the hallmark of a dark exciton ground-state. **d**–**f** Polariton dispersion relations recorded at 150, 50 and 10 K. In contrast to bare excitons, the LP luminescence significantly increases at low temperatures, behaving in the same manner as that of a bright material. **g**–**i** The zoom-in images of panels (**d**–**f**) in energy. The highlighted boxes are analysis regions of integrated intensity in Fig. 4.

Figure 3a–c shows the PL intensity distribution of the pristine WSe_2_ monolayer for three temperatures, 150, 50 and 10 K. The pump power is kept at 30 μW (see more details in “Methods” section). At 150 K, one clearly sees a strong PL signal from the flake region at the energy corresponding to the bright exciton with a broad emission tail at lower energies, which is probably associated with trions and spectrally broad defect-induced PL. As the temperature is reduced down to 50 K and finally 10 K, the exciton energy is blue-shifted by tens of meV^33^, and the corresponding emission intensity is reduced by a factor of 20 due to the fast non-radiative relaxation to the spin-forbidden dark ground state^10^. Note that also the dark exciton substantially blue-shifts upon temperature reduction.

The angle-resolved PL spectra for the WSe_2_ flake inside the microcavity at the same temperatures are presented in Fig. 3d–f. To show the polaritonic data clearly, the photon energy scale is zoomed-in in panels g–i. The LP energy position is mainly defined by the cavity mode, which is unaffected by the temperature changes, and thus the thermal blueshift of LP is severely reduced with respect to that of the bare exciton. The detailed analysis of the peak corresponding to the ground state is presented in Supplementary Fig. S4. Most interestingly, the LP photoluminescence strongly increases in intensity with decreasing temperatures: The maximal emission intensity of the polariton ground state at 10 K is four times larger than that at 150 K, while it is enhanced by seven times for the first excited polariton state in the trap. This opposite temperature dependence of PL clearly indicates that the ground state of our hybrid system becomes optically bright.

**Fig. 4:**
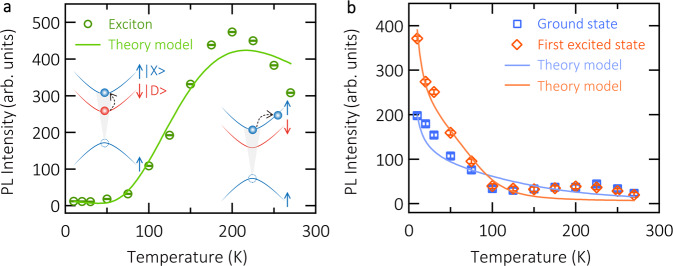
Integrated PL intensity of bare excitons and exciton-polaritons as a function of temperature. **a** Temperature-dependent emission intensity of bare excitons in pristine WSe_2_ monolayer. The experimental data are shown as green circles, and the corresponding integration regions are marked as dashed boxes in Fig. 3a–c. The solid curve represents the result of a theoretical modelling (see main text). The mechanism of PL intensity for different temperature ranges is shown as insets: thermal activation (10–200 K) and relaxation into momentum-forbidden dark states (200–270 K). **b** Temperature-dependent PL emission intensity of exciton-polaritons. The experimental data of the ground state and first excited state are shown as blue squares and red diamonds, respectively. The integration region of the ground state (first excited state) is indicated with a dashed (dotted) box in Fig. 3g–i. The solid curves are fits of the theory model. The strong PL intensity at low temperatures evidences the brightening effect of intrinsically dark exciton. The error bars are obtained by comparing the signal intensity to the standard deviation of the background noise.

In Fig. 4a, we present the integrated PL intensity from bare excitons in a pristine WSe_2_ flake as a function of temperature. The integrated areas are displayed as dashed boxes in Fig. 3a–c. As the temperature is reduced from 200 to 10 K, the PL intensity (green circles) experiences an exponential drop, stemming from the fast relaxation towards the dark ground state^10^. As shown in the left inset of Fig. 4a, the presence of dark excitons at energies that lie below bright excitons, suggests preferential population of the dark state with the reduction of temperature. The quenched light emission at low temperatures can be thermally activated following the Boltzman distribution^10^. The slight intensity reduction in the region from 200 to 270 K was previously reported in flux-grown samples^34^, and was attributed to phonon-induced non-radiative channels. The schematic is displayed as the right inset of Fig. 4a. With the increase of temperature, acoustic phonons start to participate in the scattering with bright excitons, yielding final states that are dark due to the momentum-forbidden condition. It is worth noting, that the precise trend is slightly dependent on the integration area, and there could be sample-to-sample differences, depending on the method of sample growth, exfoliation, etc, but the general phenomenon of a generic intensity reduction is universally present^10,34^.

We modelled this temperature dependence of the bare exciton PL by solving the system of rate equations corresponding to the phonon-assisted population relaxation in a pristine WSe_2_ monolayer (see details in the Supplementary section 1). In this model, two kinetic equations are considered, which represent exciton occupancies of the bright and dark states. The bright exciton scatters into dark exciton by emitting a phonon, with a rate that depends on the bare exciton-phonon scattering rate *W*_0_. The model fit is shown as a solid curve in Fig. 4a, closely reproducing the experimental trend. One clearly sees the thermal activation of the PL in the range from 10 to 200  K. Above this temperature, due to the relaxation into momentum-forbidden dark states that lie outside the light cone, the depletion of bright-exciton starts to play a role, which results in the slow decrease of PL in the range between 200 and 270 K.

The temperature dependence of the PL intensity in the strong coupling regime is presented in Fig. 4b, and clearly displays the opposite trend. The data for both trapped polariton states, ground state (blue squares) and the first excited state (red diamonds), are similarly analyzed and plotted. These polariton states show simultaneously a small intensity peak at around 225 K, which reproduces the behaviour of bare excitons. Below 150 K the polariton PL dramatically enhances with reduced temperature, clearly demonstrating the brightening effect of the polariton ground state. Furthermore, we analyze the intensity of higher energy states that range from 1.64 to 1.66 eV in Supplementary Fig. S6. Its temperature dependence exhibits the same result as the ground and first excited states in Fig. 4b. Those higher energy states lie below the dark exciton, and polaritons relax into the ground state in a dynamic, cascaded manner. As such, it is natural to encounter increase of luminescence intensity of higher polariton states.

The results of the theoretical modelling based on a system of rate equations are shown by solid curves in Fig. 4b (see the details in the Supplementary section 2) and are in good quantitative agreement with the experimental data. In the simulations, we account for the ground and first excited cavity modes, and thus consider three polariton states. The resulting system contains four equations describing the transitions between dark exciton and polariton branches (Supplementary section 2). The equation for the ground state occupancey reads:

1 $${\dot{n}}_{L}=-{n}_{L}/{\tau }_{L}+{X}_{L}^{2}{P}_{{{{{\rm{eff}}}}}}-{W}_{L\to D}({n}_{D}+1){n}_{L}+{W}_{D\to L}({n}_{L}+1){n}_{D}$$

where ${\tau }_{L}^{-1}={X}_{L}^{2}{\tau }_{nr}^{-1}+C{1}_{L}^{2}{\tau }_{c1}^{-1}+C{2}_{L}^{2}{\tau }_{c2}^{-1}$, *X*_*L*_, *C*1_*L*_, *C*2_*L*_ are the corresponding Hopfield coefficients, and *W* are the phonon-assisted inelastic scattering rates, the expressions for which are presented in the Supplementary section 2.

To account for the fast thermalization of non-resonantly pumped excitons, which leads to the decrease of the fraction of the thermal excitons lying within the light cone and forming polaritons with temperature, we introduce the effective pumping *P*_eff_ which is expressed as:

2 $${P}_{{{{{\rm{eff}}}}}}\approx P\frac{{\epsilon }_{lc}}{{k}_{B}T},$$

where *ϵ*_*lc*_ is the energy of the excitons corresponding to the wavevector of light in the material,

3 $${\epsilon }_{lc}=\frac{{\hslash }^{2}}{2{M}_{{{{{\rm{exc}}}}}}}{\varepsilon }_{{{{{\rm{Ti{O}}}}}}_{2}}{({\omega }_{x}/c)}^{2}\approx 58\,\upmu \,{{{{{\rm{eV}}}}}}\approx 0.6\,{{{{{\rm{K}}}}}}.$$

This approximation holds for *T* ≫ 0.6 K.

As lower polariton states at around *k*_∥_ = 0 have energies far below the dark exciton energy, LP can be effectively populated by inelastic processes of phonon emission. Thus, differently from the case of bare excitons, most of the bright excitons contribute to the PL signal via polariton emission at low temperatures, which explains the rapid increase of PL intensity with the decreasing temperature. In this work, we study temperature-dependent polariton emission while the Rabi-splitting remains approximately constant. Similarly, we believe that tuning of the Rabi-splitting, e.g., using an open-cavity^35^ at constant temperature could be an alternative method to probe the brightening effect.

In strongly coupled organic microcavities, the reverse intersystem crossing rates are unchanged because of the delocalization nature of polaritons^26,36^. In this work, however, we find the strong coupling is a feasible approach. Two possible reasons may be attributed: (1) the spatial confinement of polaritons, which facilitates polaritonic relaxation to the ground state via phonons, (2) the high-quality factor of optical cavity, which prolongs lifetimes of polaritons.

## Discussion

In summary, we demonstrate the possibility to effectively brighten an intrinsically dark semiconductor monolayer by placing it inside a resonantly tuned optical microcavity in the regime of strong light-matter coupling.

In our experiments, we utilize a WSe_2_ monolayer flake, which features the spin-forbidden dark exciton ground state, separated from the bright state by an energy of ~40 meV. This splitting is comparable with the vacuum Rabi-splitting characteristic to the resonant coupling of bright excitons with cavity photons, which allows to push the energy of the lower polariton below the energy of the dark exciton. As a consequence, the ground state of the system becomes bright, and instead of showing a PL quenching, characteristic for pristine WSe_2_ monolayers, we observe a strong PL enhancement with decreasing temperature, due to the fast and efficient phonon-assisted energy relaxation.

In a broader scale, our approach for band-structure engineering aligns with contemporary efforts to tune transport, topological and magnetic properties of low dimensional materials using the fundaments of cavity quantum electrodynamics.

## Methods

### Sample fabrication

The bottom DBR, composed of 10 pairs of SiO_2_/TiO_2_ layers, is obtained commercially from Laseroptics GmbH. The mechanically exfoliated WSe_2_ monolayer is capped by a ~5 nm h-BN multilayer via the deterministic dry-transfer method. The top DBR is evaporated by an ion-assisted physical vapour deposition process^37^, consisting of 9 pairs of SiO_2_/TiO_2_ layers: the SiO_2_ layer in contact with h-BN is 105 nm in thickness, and the thickness of each repetitive pair is 129 and 83 nm, respectively (more details are described in ref. ^29^). The control sample constituted by a pristine WSe_2_ monolayer is also mechanically exfoliated from a bulk crystal. It is dry-transferred onto another identical DBR. An h-BN multilayer with similar thickness ends the final capping.

### Experimental setup

A standard back Fourier plane imaging setup is utilized to perform angle-resolved PL measurements. The excitation source is a continuous-wave (CW) 532 nm laser, and it is focused on the sample by a long working-distance objective (Mitutoyo M Plan Apo NIR 50×, NA = 0.42). The charge-coupled device (CCD) of Andor (iDus 416) is attached to a spectrometer (Shamrock 500i). Its sensor is operated at −80 ^∘^C. A 600 nm longpass filter of Thorlabs (FELH0600) is used to block the green laser from reaching the CCD. In the measurements of Fig. 3a–c, the pump power is kept at 30  μW, and the exposure time is 2 s. In the measurements of Fig. 3d–f, the pump power is 30 μW and the exposure time is increased to 10 s, to compensate for the less efficient light-collection in the back-fourier plane imaging configuration. To carry out temperature-dependent experiments, the sample is mounted on a motorized XY stage of a customized low-vibration cryostat from ColdEdge. The available lowest temperature is 10 K, and the temperature of the sample is adjusted by a Lakeshore temperature controller (Model 335). The magnetic measurements are performed at room temperature using an attoDRY2100. A CW 532 nm laser excites the sample, and a set of quarter waveplate, half waveplate as well as linear polarizer in the collection path is used to detect circularly polarized PL emission. In magnetic field measurements, the used pump power is 100 μW and the exposure time is 120 s.

## Supplementary information

Supplementary Information

## Data Availability

The data that support the findings of this study are available from the corresponding authors upon reasonable request.
