# Supplementary material for: Brightening of a dark monolayer semiconductor via strong light-matter coupling in a cavity
Source: Nat Commun. 2022 May 30;13:3001. doi: 10.1038/s41467-022-30645-5 (PMC9151642; doi:10.1038/s41467-022-30645-5)
Supplement: Supplementary file 1 — Supplementary Information [file 41467_2022_30645_MOESM1_ESM.pdf]

Supplementary information: Brightening of a dark monolayer semiconductor via strong light-matter coupling in a cavity

Supplementary section 1: Model of temperature-dependent exciton photoluminescence of WSe_2 monolayer

To model the temperature dependent PL signal of the uncoupled exciton in WSe_2 monolayer, we solve the system of two kinetic equations for the exciton occupancies n_B, n_D in the bright and dark states, respectively:

$$\dot{n}_B = P_{eff} - n_B/\tau_R - n_B/\tau_{NR} - W_{B\to D} n_B(1+n_D) + W_{D\to B} n_D(1+n_B), \quad \text{(Eq. S1)}$$

$$\dot{n}_D = -n_D/\tau_{NR} + W_{B\to D} n_B(1+n_D) - W_{D\to B} n_D(1+n_B), \quad \text{(Eq. S2)}$$

where P_{eff} defines the effective incoherent pumping of the bright excitons. We assume that as pumped incoherently, the excitons thermalize very quickly, and only the excitons lying within the light cone contribute to the PL signal. For not very low temperature, the effective pumping P_{eff} is

$$P_{eff} \approx P \frac{\epsilon_{lc}}{k_B T}, \quad \text{(Eq. S3)}$$

where

$$\epsilon_{lc} = \frac{\hbar^2}{2M_{exc}} \varepsilon_{TiO_2} (\omega_x/c)^2 \approx 58\ \mu eV \approx 0.6K. \quad \text{(Eq. S4)}$$

This approximation holds for $T \gg 0.6$K. Scattering rates W correspond to the inelastic phonon-assisted process. Since dark excitonic states lie below the bright ones, the bright to dark exciton scattering occurs with the emission of a phonon, and a reverse process with an absorption of the phonon. Assuming the system is at thermal equilibrium at temperature T, the scattering rates can be expressed as:

$$W_{B\to D} = \frac{W_0}{1 - \exp[-\Delta_{BD}/k_B T]}, \quad \text{(Eq. S5)}$$

$$W_{D\to B} = W_{B\to D} e^{-\Delta_{BD}/k_B T}, \quad \text{(Eq. S6)}$$

where Δ_{BD} is the bright-dark exciton splitting which is equal to the spin splitting in the conduction band and is equal to approximately 40 meV [S1, S2], and W_0 is the bare exciton-phonon scattering rate which is a fitting parameter. The radiative exciton lifetime of the excitons within the light cone is set to 4 ps which corresponds to previously reported theoretical and experimental

values [S3, S4] and the non-radiative lifetime which is typically related to the defect trapping is set to $\tau_{NR} = 5$ ps [S5]. By fitting the experimental temperature dependence we obtain the value of $W_0 \approx 10$ ps^{-1}.

Supplementary section 2: Model of ground state brightening via strong light-matter coupling

We consider a simplified picture, where there is a single exciton state, and two cavity modes, corresponding to the ground and excited exciton modes in the trap (Supplementary Fig. S1). We can write down the coherent part of the Hamiltonian in the matrix form

$$H_{coh} = \begin{pmatrix} b^\dagger & a_1^\dagger & a_2^\dagger \end{pmatrix} \begin{pmatrix} \omega_x & g_1 & g_2 \\ g_1 & \omega_{c1} & 0 \\ g_2 & 0 & \omega_{c2} \end{pmatrix} \begin{pmatrix} b \\ a_1 \\ a_2 \end{pmatrix} \qquad \text{(Eq. S7)}$$

where b, a_1, a_2 are the annihilation operators for the exciton and two cavity modes. The frequencies ω_x, ω_{c1}, ω_{c2} can be extracted from the experimental data, and g_1, g_2 are the fitting parameters in our model.

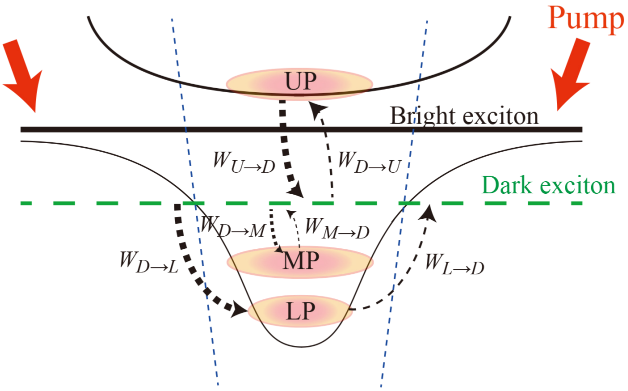

Supplementary Fig. S1 Scheme of phonon-assisted population relaxation of the hybrid system. In the simulations, we consider four population equations for the dark excitons as well as three polariton branches LP, MP and UP. The expressions of scattering rates of corresponding transitions are written as Supplementary Eqs. (S8-13).

This Hamiltonian can be diagonalized yielding lower, middle and upper polariton modes a_L, a_M, a_U. Moreover, we obtain the polariton energies $\epsilon_L, \epsilon_M, \epsilon_U$ and exciton fraction in each of the polaritonic modes (Hopfield coefficients): X_U, X_M, X_L. The rate of the phonon assisted scattering between the polaritons and the spin-dark excitons D is proportional to the exciton fraction in the respective polariton, and the occupation number of the phonons at the

energy corresponding to the energy difference between the dark exciton and respective polariton. If the scattering occurs with the emission of phonon, the rate is proportional to $n_{ph} + 1$ and if the scattering is with the absorption of phonon, then the rate is proportional to n_{ph}. The phonon scattering rates thus are:

$$W_{U\to D} = X_U^2 W_0 \frac{1}{1-\exp[-(\epsilon_U - \epsilon_D)/(k_B T)]}, \qquad \text{(Eq. S8)}$$

$$W_{D\to U} = e^{-(\epsilon_U - \epsilon_D)/(k_B T)} W_{U\to D}, \qquad \text{(Eq. S9)}$$

$$W_{D\to M} = X_M^2 W_0 \frac{1}{1-\exp[-(\epsilon_D - \epsilon_U)/(k_B T)]}, \qquad \text{(Eq. S10)}$$

$$W_{M\to D} = e^{-(\epsilon_D - \epsilon_U)/(k_B T)} W_{D\to M}, \qquad \text{(Eq. S11)}$$

$$W_{D\to L} = X_L^2 W_0 \frac{1}{1-\exp[-(\epsilon_D - \epsilon_L)/(k_B T)]}, \qquad \text{(Eq. S12)}$$

$$W_{L\to D} = e^{-(\epsilon_D - \epsilon_L)/(k_B T)} W_{D\to L}, \qquad \text{(Eq. S13)}$$

where W_0 is the bare rate of exciton-phonon scattering obtained via fitting of the bare exciton PL signal. We also need to account for the fact that at non-resonant pumping only the excitons with the wavevectors inside the light cone form the polaritons. The system of equation then reads

$$\dot{n}_U = -n_U/\tau_U + X_U^2 P_{eff} - W_{U\to D}(n_D+1)(n_U) + W_{D\to U} n_D (n_U+1), \qquad \text{(Eq. S14)}$$

$$\dot{n}_M = -n_M/\tau_M + X_M^2 P_{eff} - W_{M\to D}(n_D+1)n_M + W_{D\to M}(n_M+1)n_D, \qquad \text{(Eq. S15)}$$

$$\dot{n}_L = -n_L/\tau_L + X_L^2 P_{eff} - W_{L\to D}(n_D+1)n_L + W_{D\to L}(n_L+1)n_D, \qquad \text{(Eq. S16)}$$

$$\dot{n}_D = -n_D/\tau_D + W_{U\to d}(n_D+1)(n_U) - W_{D\to U} n_D (n_U+1) + W_{M\to D}(n_D+1)n_M - \\ - W_{D\to M}(n_M+1)n_D + W_{L\to D}(n_D+1)n_L - W_{D\to L}(n_L+1)n_D \qquad \text{(Eq. S17)}$$

where $\tau_{U,M,L}^{-1} = X_{U,M,L}^2 \tau_x^{-1} + C1_{U,M,L}^2 \tau_{c1}^{-1} + C2_{U,M,L}^2 \tau_{c2}^{-1}$, and τ_D is the dark exciton lifetime which is set equal to the non-radiative exciton lifetime. The values of $g_1 = 0.02$ eV and $g_2 = 0.016$ eV are extracted from fitting the experimental temperature dependence of the polariton PL signal. Moreover, in fitting we accounted for the temperature dependence of the bare exciton energy $\omega_x \approx 1.73 - 0.06(T/300)$ eV.

Supplementary section 3: Polariton dispersion relations versus air pressure

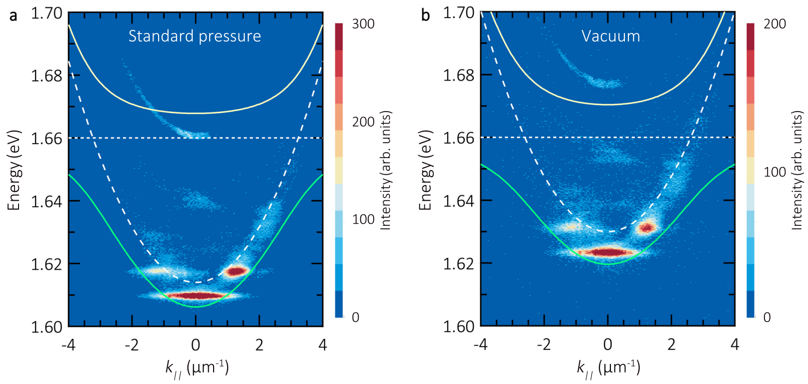

Supplementary Fig. S2 Dispersion relations of room temperature polaritons at standard pressure and high vacuum. a Polariton dispersion relation at ambient conditions, which is reproduced from Fig. 2a in the main text. The bare cavity mode is at 1.614 eV. **b** Polariton dispersion relation at room temperature and high vacuum. The cavity mode is changed to 1.630 eV as the pressure is decreased to 3E-5 hPa.

Air pressure can affect the energy of microcavity photons and LP. Supplementary Fig. S2a shows the polariton dispersion at ambient conditions (room temperature, standard pressure), which is reproduced from Fig. 2a in the main text. The cavity mode is at 1.614 eV, and the energy of ground state is 1.610 eV.

Weak emission detected above 1.66 eV is collected from areas without the monolayer-hBN stack, i.e. the barrier of the polariton trap (the collection area of PL is tens of microns). Since the optical length of the cavity is significantly reduced at those positions, it occurs at strongly elevated energy. In the barrier, there is no active material which can directly generate luminescence, however light, which is generated by the polaritons in the trap or which is generated by weakly coupled excitons on the lateral interface between trap and barrier, can scatter into these modes.

Before performing temperature-dependent measurements, the cryostat is pumped several hours to get a high vacuum. Supplementary Fig. S2b shows the dispersion relation after vacuum pump (room temperature, pressure of 3E-5 hPa). We observe an energy shift of LP, which originates from the alteration of cavity mode. The cavity photon energy is changed to 1.630 eV and the resulted LP is at 1.624 eV. This energy shift is reversible: the dispersion relation gets back after measurements, when the air pressure is recovered.

Supplementary section 4:
The valley-Zeeman effect at low pump power

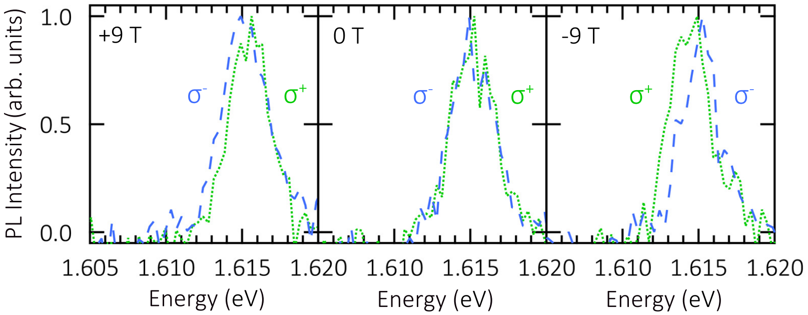

Supplementary Fig. S3 Normalized circularly-polarized PL intensity of exciton-polaritons under a magnetic field at room temperature. The pump power is 10 μW. From left to right panels, the applied magnetic field is +9, 0 and -9 T. σ^+ (σ^-) denotes emission light of right (left)-hand circular polarization.

Supplementary section 5:
Supplied temperature dependent data of exciton-polaritons

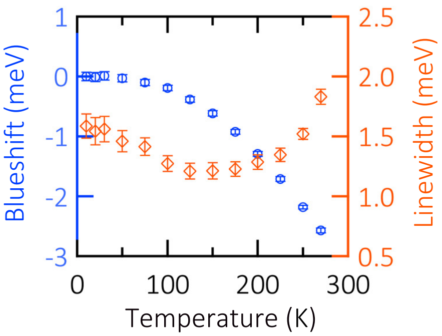

Supplementary Fig. S4 Blueshift (blue circles) and linewidth (red diamonds) of polariton ground state as a function of temperature. The energy of polariton ground state is blueshifted ~3 meV cooling from 270 K to 10 K. The error bars of blueshift and linewidth correspond to the 95% confidence interval of the peak fitting.

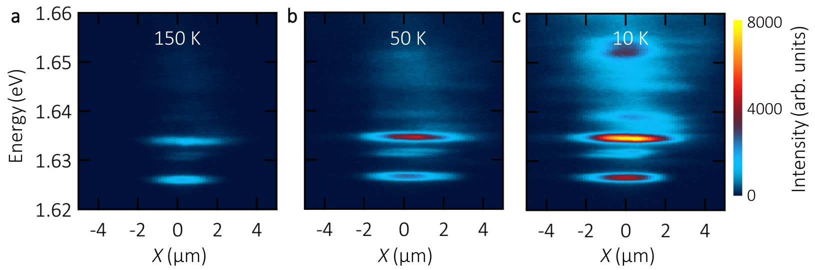

Supplementary Fig. S5 Real-space resolved PL intensity distribution of polaritons as a function of energy. a-c The temperature is 150 K, 50 K and 10 K. The emission intensity of polaritons in real-space agrees with the trend of momentum-space as shown in Figs. 3d-f in the main text.

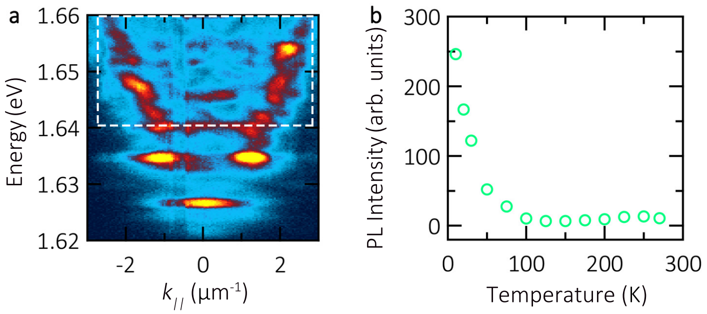

Supplementary Fig. S6 Integrated PL intensity of polaritons. a is reproduced from Fig. 3i in the main text. The white box represents the integration region that ranges from 1.64 to 1.66 eV. **b** Temperature dependent emission intensity of polaritons, with the integration region shown in a.

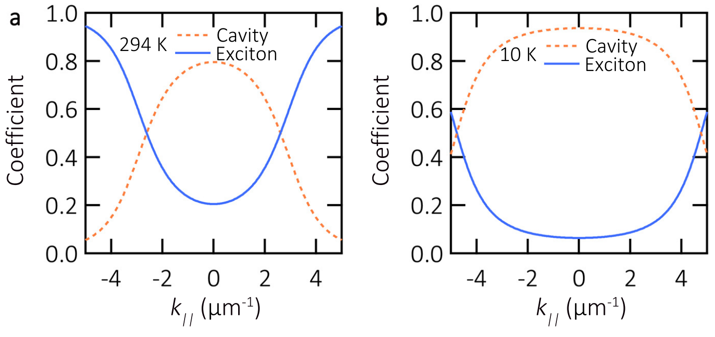

Supplementary Fig. S7 Hopfield coefficients of LP at temperatures of 294 K (a) and 10 K (b). The exciton fraction of LP is 20.5% at 294 K, and it decreases to 6.4% as the temperature is 10 K.

Supplementary section 6: Supplied temperature dependent data of pristine WSe_2 monolayer

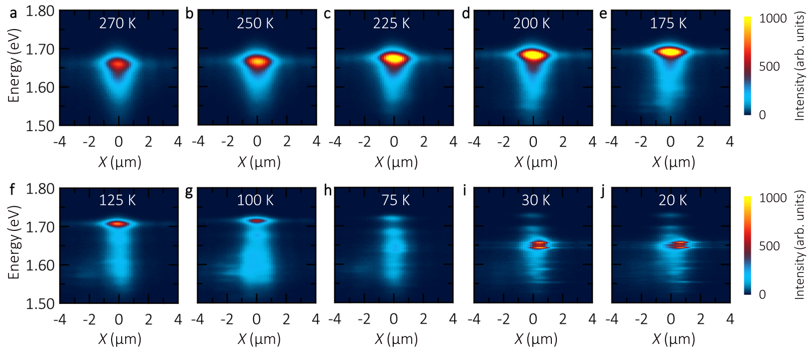

Supplementary Fig. S8 Real-space resolved PL intensity distribution of pristine WSe_2 **monolayer as a function of energy.** The temperature decreases from 270 K to 20 K. The rest data of 150 K, 50 K and 10 K are shown in Figs. 3a-c in the main text.

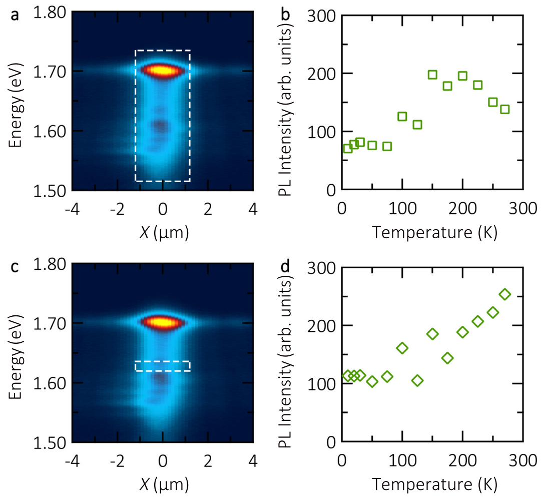

Supplementary Fig. S9 Integrated PL intensity of pristine WSe_2 **monolayer. a** is reproduced from Fig. 3a in the main text. The white box represents the integration region that ranges from 1.52 to 1.74 eV (including all emission features). **b** Temperature dependent emission intensity of pristine WSe_2 monolayer, with the integration region shown in a. **c** The integration region ranges from 1.623 to 1.636 eV, corresponding to energies of ground state and first excited state of polaritons. **d** Temperature dependent emission intensity of pristine WSe_2 monolayer, with the integration region shown in c.

Supplementary references

[S1] Zhou, Y. *et al.* Probing dark excitons in atomically thin semiconductors via near-field coupling to surface plasmon polaritons. *Nature Nanotechnology* **12** (9), 856–860 (2017) .

[S2] Wang, G. *et al.* In-plane propagation of light in transition metal dichalcogenide monolayers: Optical selection rules. *Physical Review Letters* **119** (4), 047401 (2017) .

[S3] Wang, G. *et al.* Valley dynamics probed through charged and neutral exciton emission in monolayer WSe_2. *Physical Review B* **90** (7), 075413 (2014) .

[S4] Palummo, M., Bernardi, M. & Grossman, J. C. Exciton radiative lifetimes in two-dimensional transition metal dichalcogenides. *Nano Letters* **15** (5), 2794–2800 (2015) .

[S5] Korn, T., Heydrich, S., Hirmer, M., Schmutzler, J. & Schüller, C. Low-temperature photocarrier dynamics in monolayer MoS_2. *Applied Physics Letters* **99** (10), 102109 (2011) .
